# *Suan Zao Ren Tang* in Combination with *Zhi Zi Chi Tang* as a Treatment Protocol for Insomniacs with Anxiety: A Randomized Parallel-Controlled Trial

**DOI:** 10.1155/2015/913252

**Published:** 2015-02-22

**Authors:** Lin-lin Hu, Xin Zhang, Wen-juan Liu, Mei Li, Yong-hua Zhang

**Affiliations:** ^1^School of Traditional Chinese Medicine, Zhejiang Chinese Medicine University, Zhejiang 310053, China; ^2^School of Traditional Chinese Medicine, Heilongjiang University of Chinese Medicine, Heilongjiang 150040, China; ^3^Department of Medicine, The Seventh Hospital of Hangzhou, Mental Health Care of Hangzhou, Zhejiang 310013, China; ^4^School of Medicine, Anhui Medical University, Anhui 230032, China

## Abstract

Insomnia is a serious worldwide health problem that is often comorbid with anxiety. The purpose of the present study was to evaluate the efficacy of a Chinese formula containing *Suan Zao Ren Tang* (SZRT) and * Zhi Zi Chi Tang* (ZZCT; SZR-ZZC) for improving sleep quality and anxiety states with four indices of Polysomnography (PSG), the Insomnia Severity Index (ISI), the Pittsburgh Sleep Quality Index (PSQI), and the Self Rating Anxiety Scale (SAS). * Methods.* A randomized, parallel-controlled trial compared SZR-ZZC to lorazepam tablet in insomniacs with anxiety. Patients were randomized to the SZR-ZZC treatment group (*n* = 60) and the lorazepam tablet treatment group (*n* = 59). *Results*. SZR-ZZC significantly improved scores on all four treatment indices. Compared with lorazepam, treatment with SZR-ZZC resulted in a significant reduction in the ISI (*P* = 0.029), the PSQI (*P* = 0.017), and wake after sleep onset (WASO; *P* = 0.008) scores and improved sleep architecture (*P* = 0.000–0.003) after a 4-week treatment period. Only one subject in the SZR-ZZC group experienced adverse side effects. *Conclusion*. Treatment with SZR-ZZC for 4 weeks appears to be a relatively safe and effective complementary therapeutic option when aiming to improve sleep quality and anxiety in insomniacs with anxiety.

## 1. Introduction

Insomnia not only is one of the most prevalent sleep disorders among the general population, but also presents a significant challenge to the maintenance of one's health. In 2002, approximately 10% of the world's population suffered from chronic or persistent insomnia [1, 2]. Additionally, insomnia patients typically suffer from comorbid emotional issues including depression, anxiety, nervousness, and irritability [3]. In fact, 54% of primary insomnia patients have a coexisting emotional disorder [4].

The conventional treatments for insomnia include GABA-A receptor modulators and histamine receptor antagonists, but if an insomnia patient presents with anxiety, an anxiolytic or antidepressant drug may also be administered [5]. In general, most pharmacotherapies have similar effects on objective measures of sleep latency, but when measured objectively, some drugs are more effective and others less effective [6]. Moreover, the adverse effects (AEs) associated with these drugs are often overexaggerated in insomniacs with anxiety, and the abuse of benzodiazepines is a serious problem in some countries, including those in North America [7]. These issues may motivate patients to seek remedies from traditional Chinese medicine (TCM). TCM is used extensively in China, and even Southeast Asia, because it is readily available, culturally acceptable, and effective. For example, the prevalence of the concurrent use of sedative-hypnotics and Chinese herbal products is 37% among the Taiwanese population [8].

The formula used in present study containing* Suan Zao Ren Tang* (SZRT) and* Zhi Zi Chi Tang* (ZZCT). SZRT and ZZCT have been used to treat insomnia separately for centuries. They were first documented by* Zhong-jing Zhang* in approximately 210 A.D. in the classic Chinese text* Jin Gui Yao Lve* (Synopsis of Prescriptions of the Golden Chamber). SZRT is a blend of five medicinal Chinese herbs including* Semen Zizyphi Spinosae* (*Suanzaoren*),* Sclerotium Poriae Cocos* (*Fuling*),* Radix Ligustici Chuanxiong* (*Chuanxiong*),* Rhizoma Anemarrhena* (*Zhimu*), and* Radix Glycyrrhizae* (*Gancao*), while ZZCT is a combination of the two following dried raw materials from* Gardenia Jasminoides fruit* (*Zhi zi*) and* fermented soybeans* (*Dandouchi*; Table 1). According to classic theory, SZRT is beneficial for the replenishment of liver-yin and kidney-yin, while ZZCT aids in eliminating internal fire. Together these two classic compounds cooperate to nourish yin and clear fire.

A majority of insomnia patients with anxiety may be described as having a yin-deficiency and fire-excess syndrome which in clinical terms often manifest as insomnia accompanied with anxiety, palpitations, restlessness, headaches, red tongue, and a rapid pulse [9]. In TCM, a formula containing SZRT in combination with ZZCT (SZR-ZZC) is thought to be suitable for this condition. A previous study from our research group found encouraging results following treatment with SZR-ZZC [9]; however, despite the popular use of SZRT and ZZCT, there is little scientific evidence supporting the efficacy of the clinical use of SZR-ZZC. Thus, the present study was a 4-week, randomized, parallel-controlled trial conducted to verify the effects and safety of SZR-ZZC for the treatment of insomniacs with anxiety.

## 2. Materials and Methods

### 2.1. Medicinal Preparations

All herbal components of the SZR-ZZC formula were prepared according to traditional methods in the proportions listed in Table 1. The herbal broth was dispensed into 150 mL packages, and each package of decoction was made from 38.5 g crude drugs. In order to ensure the quality and uniformity of the herbs and decoctions, all herbs were provided by a certified company (Medicinal Materials Co. Ltd; Lin'an City, Zhejiang Province, China), which was not involved in the sponsorship, or design of the study, or monitoring of the participants. Lorazepam tablets (0.5 mg; Atlantic Laboratories Corporation Ltd; Thailand) were purchased in boxes of 20 for use as the control drug.

### 2.2. Recruitment of Subjects

All subjects enrolled in the present study were recruited from the Seventh Hospital of Hangzhou between March 2013 and March 2014. All subjects provided written informed consent prior to participation in the study, and the subjects were free to withdraw at any time. The trial protocol was approved by the Ethics Committee of The Seventh Hospital of Hangzhou.

### 2.3. Randomization and Blinding Procedures

All eligible subjects were placed into either the SZR-ZZC treatment group or the parallel control group using random numbers generated by a computer software program. The treatment codes were safeguarded by the chief investigator, who was isolated from all subjects and the outcome data. The SZR-ZZC and lorazepam treatments were distributed to the subjects by the pharmacy staff with assistance from our research staff, and all subjects, pharmacy staff, research staff, and data entry clerks were blinded to the treatment groups. Treatment compliance was ensured by the research staff and the treatment codes were disclosed after the entire study was completed.

### 2.4. Study Design and Setting

All subjects underwent a preliminary screening for nonorganic insomnia according to the criteria of 10th revision of the International Classification of Diseases (ICD-10; F51.0) and performed by the researchers of this study (deputy director of the physician and above). Additionally, each subject was interviewed regarding their medical history and completed a clinical examination that included the Self-Rating Anxiety Scale (SAS), Pittsburgh Sleep Quality Index (PSQI), and Insomnia Severity Index (ISI).

The inclusion criteria for the present study were patients between 18 and 60 years of age, who struggled with insomnia for more than 6 months, who met the ICD-10 criteria for nonorganic insomnia, and who had an SAS score > 50, a PSQI score > 6, and an ISI score > 7. The exclusion criteria consisted of working nightshift; a past or current history of schizophrenia, bipolar, or other psychotic disorders; the presence of confounding diseases such as obstructive sleep apnea-hypopnea, restless leg syndrome, cardiac arrhythmia, acute myocardial infarction, or cancer; the use of pharmaceuticals within the previous 1 month that could affect melatonin, acetylcholine, glutamate, serotonin, norepinephrine, GABA, histamine, adenosine, or prostaglandins, and gestating or lactating females.

**Figure 1:**
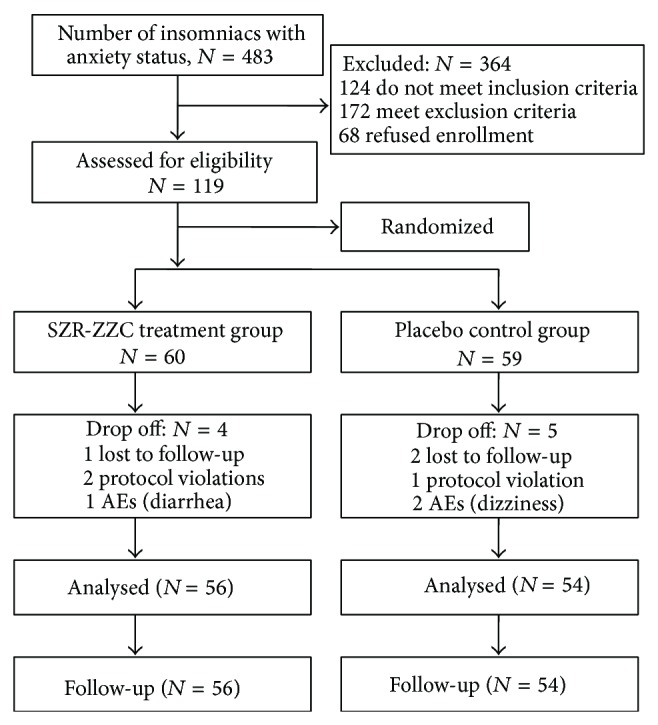
Flow chart of the study population.

All subjects who were in compliance with the inclusion and exclusion criteria were enrolled in the present study (Figure 1) and underwent a polysomnography (PSG) procedure to obtain subjective sleep quality measures.

### 2.5. Study Interventions

Subjects in the SZR-ZZC group received packages of the SZR-ZZC decoction and were instructed to drink one bag twice per day (half an hour after breakfast and supper). Subjects in the control group were instructed to take two lorazepam tablets orally twice a day (half an hour after breakfast and before bedtime).

### 2.6. Outcome Measures

The primary outcome parameters of the present study were the ISI and SAS scores, which were evaluated at baseline and after 2 and 4 weeks of treatment intervention. The secondary outcome parameters were the PSQI scores and PSG measures, which were completed at baseline and after treatment.

The ISI is a self-report measure that has been used by a number of research teams over the past 20 years [10]. It is a seven-item scale that assesses the perceived severity of insomnia symptoms using the following criteria: (a) the severity of sleep-onset (initial), (b) sleep maintenance (middle), (c) early morning awakening (terminal) problems, (d) satisfaction with current sleep pattern, (e) interference with daily functioning, (f) noticeability of impairments attributed to the sleep problem, and (g) level of distress caused by the sleep problems [11]. The typical time frame for responses on the ISI includes the most recent 2 weeks, and it is scored using a Likert-type rating system with five anchor points ranging from 0 to 4. The ISI questionnaire has been confirmed as reliable and valid for the quantification of the perceived severity of insomnia [11–14].

The SAS is a self-completed 20-item questionnaire comprised of four parts: (1) anxiety and panic (items 1–4 and 20); (2) vestibular sensations (items 6, 10–12, and 18); (3) somatic control (items 5, 9, 14, 17, and 19); and (4) gastrointestinal/muscular sensations (items 7-8, 13, and 15-16). Scores for each item range from 1 to 4 and the total of these scores is multiplied by 1.25 to obtain a standardized score (SS). The SAS has been widely used for the assessment of different populations [15, 16]. In accordance with the Chinese norms, a SS ≥ 50 indicates the presence of anxiety such that a SS = 50–59 indicates mild anxiety, a SS = 60–69 indicates moderate anxiety, and a SS ≥ 70 indicates severe anxiety.

The PSQI is a self-rated questionnaire that assesses sleep quality and disturbances over a 1-month time interval and probes clinically important and patient-relevant symptoms in the areas of sleep quality and quantity. The PSQI consists of 19 self-rated items and five other-rated items. Of these 24 items, the scores of the 19th self-rated items and five other-rated items are not included in the total scores. The remaining 18 items are divided into seven subscales: subjective sleep quality, sleep latency, sleep duration, habitual sleep efficiency, sleep disturbances, use of sleeping medication, and daytime dysfunction [17]. Each subscale is rated from 0 to 3 with a higher score indicating a more severe sleep complaint. The summed score of seven subscales yields a single global score that represents the patient's overall sleep experience; a lower total score reflects a better quality of sleep. The PSQI possesses a high test-retest reliability and good validity for insomniacs [18].

PSG is the gold standard when assessing sleep quality, but it is used infrequently for research purposes due to its complexity and expense. In the present study, the PSG analyses were performed using the Sandman system and Embla N7000 60 recording system (Medcare Flaga; Iceland). The sleep stages were scored according to the criteria of Rechtschaffen and Kales [14] based on total sleep time (TST), latency to onset of persistent sleep (LPS), and wake after sleep onset (WASO) which were calculated as follows: TST, the time spent in all sleep stages; LPS, the duration of time measured from lights off to the first of 20 consecutive epochs of the sleep stages (stages 1, 2, 3, and 4 or rapid eye movement (REM)); and WASO, the duration of the wake stage after sleep onset.

Throughout the trial, the subjects were monitored closely for adverse events (AEs) and a worsening of symptoms based on data from a blood cell analysis, urinalysis, and liver and kidney function tests, as well as patient complaints. If observed, the duration and severity of the symptoms, any action taken to alleviate these symptoms, and the outcomes were recorded.

### 2.7. Statistical Analysis

The quantitative data are summarized using means ± standard deviations (SD) or 95% confidence intervals (CI). The qualitative data are described as proportions (as percentages), and the baseline characteristics of the two groups were compared using a two-sided Chi-squared test or a *t*-test with a significance level of < 0.05 considered to indicate statistical significance.

The present analyses evaluated treatment effects according to the primary and secondary outcomes. The primary outcomes were changes in the mean differences of the ISI and SAS scores between the two groups at weeks 2 and 4. The secondary outcomes were subanalyses of the changes in scores from baseline to endpoint for each scored item on the PSQI, as well as changes in the PSG sleep parameters (TST, LPS, and WASO) and sleep architecture (slow wave sleep (SWS), REM) from baseline to endpoint. The statistical analyses for each outcome were performed by intention-to-treats (ITT) analysis using the SPSS 16.0 software package. Any missing values were imputed using the last-observation-carried-forward approach. Two-sample *t*-tests were used for intergroup comparisons, paired *t*-tests were used for intragroup comparisons, and a *P* value < 0.05 or less was considered to indicate statistical significance.

## 3. Results

### 3.1. Study Population

The present study included 483 patients who were diagnosed with nonorganic insomnia coupled with anxiety. Of these initial 483 patients 364 were excluded from and 119 were enrolled in the present trial. The principal reasons for dismissal from the study were meeting the exclusion criteria (*n* = 172), not meeting the inclusion criteria (*n* = 124), and not having interest in participation (*n* = 68). The 119 enrolled subjects were assigned randomly to either the SZR-ZZC treatment group (*n* = 60) or the lorazepam tablet control group (*n* = 59). A total of 56 of SZR-ZZC subjects (93%) and 54 lorazepam subjects (92%) completed the entire 4-week study; the reasons for withdrawal are listed in Figure 1.

The randomization procedure was effective and that there were no significant differences between the two groups. The mean age of the subjects was 45 years (range: 18–60 years), and females comprised 67% of the SZR-ZZC group and 68% of the lorazepam group.

### 3.2. Safety and AEs

Three patients withdrew from the study due to AEs: one case of diarrhoea in the SZR-ZZC group and two cases of dizziness in the lorazepam group. Further discussion regarding the possibility of diarrhoea caused by the SZR-ZZC formula is provided in Section 4.

### 3.3. Efficacy

**Figure 2:**
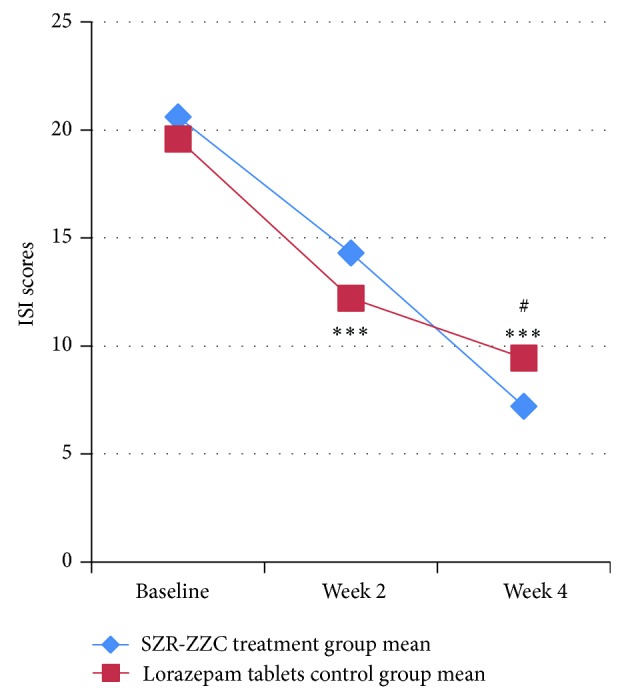
Treatment effects SZR-ZZC versus lorazepam on ISI scores at week 2 and 4. (Statistical analysis was performed using intent-to-treat (ITT) analysis with SPSS. ^#^ *P* < 0.05 according to a two-sample *t*-test comparing the two groups in week 4. ^***^ *P* < 0.001 according to a paired *t*-test comparing baseline versus week 2 and week 2 versus week 4.)

**Figure 3:**
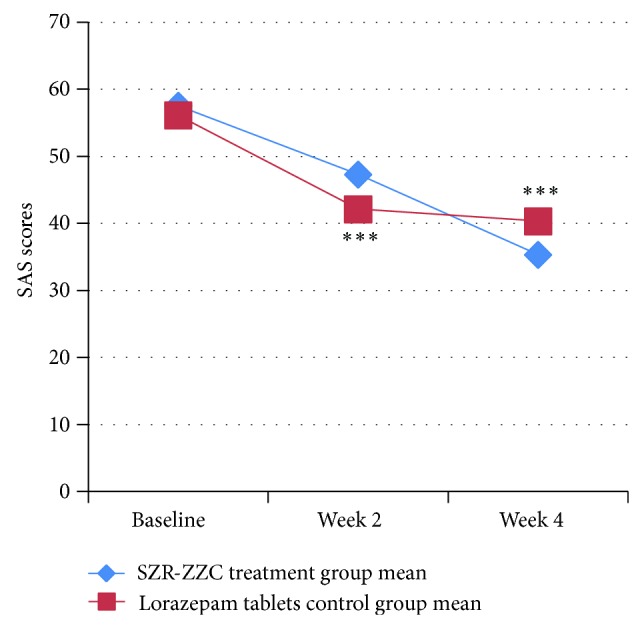
Treatment effects of SZR-ZZC versus lorazepam on SAS scores at weeks 2 and 4. (Statistical analysis was performed using intent-to-treat (ITT) analysis with SPSS. There were no significant differences between two groups at weeks 2 and 4 according to a two-sample *t*-test. ^***^ *P* < 0.001 according to a paired *t*-test comparing baseline versus week 2 and week 2 versus week 4.)

Subjects in SZR-ZZC group exhibited a significant reduction in ISI scores at weeks 2 and 4 of the treatment period (Figure 2 and Table 2). Additionally, the SZR-ZZC formula reduced ISI scores more effectively than did lorazepam tablets (*P* = 0.029). The SAS scores of subjects treated with SZR-ZZC were also decreased at weeks 2 and 4 (Figure 3 and Table 2).

Except for the daytime dysfunction subscale (*P* = 0.051), there were significant improvements on each of the PSG parameters and PSQI subscales after 4 weeks of treatment (Table 2). Additionally, the present findings demonstrate that SZR-ZZC had a greater benefit on WASO (*P* = 0.008), SWS (*P* < 0.001), REM (*P* = 0.003), and habitual sleep efficiency (*P* = 0.049).

### 3.4. Treatment Acceptability

Overall, the SZR-ZZC formula was well accepted. Of the 68 patients who expressed reluctance to participate in the present study, 45 were unwilling to take the lorazepam tablets.

## 4. Discussion

To the best of our knowledge, the present study is the first to use the SZR-ZZC formulation (SZRT + ZZCT) to treat insomnia patients with anxiety. Insomnia is frequently comorbid with anxiety [3], and the present authors have observed that the use of hypnotics is common among insomnia patients and that antidepressant drugs are prescribed in cases of insomnia with moderate to severe anxiety. However, hypnotics can only be used for a short duration, and in our opinion, it is unacceptable to take antidepressants for long periods of time. Insomniac patients often refuse antidepressants due to the likelihood of AEs, and a fear of the sedative side effects of hypnotics of the possibility of dependency may motivate these patients to seek TCM therapies [19–21]. According to TCM, blood is stored in the liver when people sleep and is an essential ingredient of body's yin. Sleep disorders lasting longer than 6 months can easily exhaust the body's yin, and a yin deficiency leads to a relatively excess of yang; meanwhile, anxious patients experience symptoms such as irritability and dysphoria, which are considered an interior sthenia-heat (fire) syndrome. Heat can also consume the body's yin. Based on the above theory, insomniacs with anxiety are experiencing yin deficiency and yang excess syndrome.

In the present study, lorazepam tablets were used as the control drug, because the subjects were insomniacs with anxiety. The present findings suggest that SZR-ZZC improves sleep quality and that lorazepam may improve the anxiety status and global sleep quality of insomnia patients (Figures 2 and 3 and Table 2). Although the similar changes in ISI scores between the groups indicate that the two approaches had an equivalent efficacy on subjective sleep after 2 weeks of treatment, changes in the subjective (ISI and PSQI scores) and objective (WASO, SWS, and REM) sleep measures indicated that SZR-ZZC had a greater benefit on insomnia patients after 4 weeks of treatment compared with lorazepam. Previous research has shown that sedative-hypnotics increase TST and shorten LPS time but also alter sleep architecture via a decrease in SWS [22]. These findings may be linked to drug tolerance issues. Regarding anxiety, the efficacies of the two treatment protocols were not significantly different, but because the SZR-ZZC formula had a more powerful effect on anxiety after 2 weeks of treatment (Figure 3), the effects will likely be similar after 4 weeks of treatment. Nonetheless, the treatment efficacies of the two groups appear to be similar regarding objective sleep outcomes.

The subjects had to withdraw from the SZR-ZZC treatment group due to AEs, primarily diarrhoea. After careful consideration of each case, the possibility that SZR-ZZC caused diarrhoea could not be disproven, and thus, one patient withdrew from the study, and one patient was treated subsequently with a modified SZR-ZZC formula. Therefore, TCM practitioners must be alert to the possibility that SZR-ZZC can cause adverse gastrointestinal effects if misused. The choice of treatment based on the differentiation of syndromes is an important feature of TCM theory, and thus, when a patient is prone to gastrointestinal issues or is spleen or stomach deficiency, TCM can prevent or cure these issues easily [23, 24].

The mechanisms underlying the efficacy of SZR-ZZC have yet to be fully characterized, but those of SZRT have been investigated by a number of studies. SZRT is very familiar to TCM practitioners, and there is a large amount of evidence supporting its beneficial effects on insomnia [25, 26]. Due to its affinity for serotonin receptors, the therapeutic effects of SZRT are likely mediated via serotonergic activation [27].* Suan Zao Ren* is the most frequently used single herb for insomnia [28], but jujubogenin which activates GABA-A receptors is also a constituent effective for the treatment of insomnia [29]. Although ZZCT has also has been widely used for the treatment of insomnia and other psychiatric diseases, its specific mechanism of action remains unclear [30]. Based on TCM theory, SZRT and ZZCT exert a synergistic action that is beneficial for insomniacs with anxiety, but the herbal interactions of the formula have yet to be investigated systematically. Further investigation is warranted to elucidate the mechanisms of action underlying SZR-ZZC.

This study possesses several limitations. First, the present study was conducted for only 4 weeks largely because the long-term use of lorazepam tablets may result in a tolerance to or dependency on the drug. Thus, it was determined that a 4-week treatment course was sufficient to observe its efficacy. Second, no long-term follow-up assessments were performed. In fact, after the 4-week treatment period, a majority of the participants shifted to consolidation therapy and no longer adhered strictly to the study protocol. Third, the present study was an exploratory trial which did not follow strict clinical practices and thus a more pragmatic trial is necessary to conform to TCM principles fully. In this case, the effects of a treatment intervention will more closely mirror those of a real clinical situation than findings from an exploratory trial [31].

## 5. Conclusions

The present findings provide preliminary support for use of the SZR-ZZC formula to treat insomniacs with anxiety. SZR-ZZC appears to be a well-accepted and valuable alternative therapeutic option for improving sleep quality and anxiety. Further studies evaluating the safety and underlying mechanisms of action of SZR-ZZC are warranted.

## Figures and Tables

**Table 1:** Nomenclature of the Chinese herbs in *Suan  Zao  Ren  Tang* and *Zhi  Zi  Chi  Tang* formula.

Pharmaceutical name	Chinese Pinyin	Latin botanical name	Proportion (%)
*Semen Zizyphi Spinosae *	*Suanzaoren *	*Ziziphus jujuba Mill. var. Spinosa *	19.5
*Sclerotium Poriae Cocos *	*Fuling *	*Poria cocos (Schw.) Wolf *	19.5
*Radix Ligustici Chuanxiong *	*Chuangxiong *	*Ligusticum chuanxiong Hort. *	13.0
*Rhizoma Anemarrhena *	*Zhimu *	*Anemarrhena asphodeloides Bge. *	15.6
*Gardenia Jasminoides fruit *	*Zhizi *	*Gardenia jasminoides Ellis *	13.0
*Fermented Soybean *	*Dandouchi *	*Semen Sojae Praepatum *	13.0
*Radix Glycyrrhizae *	*Gancao *	*Glycyrrhiza uralensis Fisch. *	6.5

**Table 2:** Treatment effects of SZR-ZZC versus lorazepam on the study variables^(1)^.

Variables	SZR-ZZC (*N* = 60)		Paired t-test^(2)^ mean difference (95% CI)	Lorazepam tablets (*N* = 59)		Two sample t-test^ (3)^ mean difference (95% CI)
	Baseline mean (SD)	Week 4 mean (SD)		Baseline mean (SD)	Week 4 mean (SD)	
PSG (min)						
TST	362.6 (37.1)	450.7 (25.7)	(−103.33, −72.81)^*^	352.6 (35.2)	456.4 (37.8)	(−22.53, 11.10)
LPS	48.7 (24.31)	24.5 (14.29)	(15.17, 33.22)^*^	45.2 (26.07)	19.2 (16.42)	(−2.72, 13.31)
WASO	92.1 (23.02)	17.9 (14.58)	(63.12, 85.42)^*^	95.9 (20.59)	31.9 (23.98)	(−24.37, 13.76)^**^
SWS	30.6 (13.66)	75.0 (12.36)	(−51.55, −37.25)^*^	26.1 (12.53)	62.3 (10.82)	(6.62, 18.75)^*^
REM	36.7 (14.92)	84.7 (15.73)	(−55.97, 40.03)^*^	34.4 (14.75)	71.8 (16.64)	(4.50, 21.38)^**^
ISI (scores)	20.6 (4.10)	7.2 (3.60)	(10.62, 15.38)^***^	19.6 (4.15)	9.4 (4.11)	(−4.26, −0.23)^*^
SAS (scores)	57.5 (6.38)	35.3 (12.90)	(16.27, 28.13)^***^	56.1 (4.93)	40.3 (10.48)	(−12.67, 2.63)
PSQI (scores)	14.2 (1.93)	7.6 (3.84)	(4.33, 8.87)^***^	13.9 (2.42)	12.0 (3.35)	(−7.91, −0.89)^*^
Subjective sleep quality	2.5 (0.53)	1.3 (0.67)	(0.64, 1.76)^***^	2.6 (0.53)	1.4 (1.13)	(−1.03, 0.75)
Sleep latency	2.7 (0.48)	1.8 (0.79)	(0.37, 1.43)^**^	2.7 (0.50)	1.7 (0.71)	(−0.60, 0.86)
Sleep duration	2.7 (0.48)	1.4 (1.17)	(0.62, 1.98)^**^	2.4 (0.53)	1.3 (0.87)	(−0.94, 1.08)
Habitual sleep efficiency	2.9 (0.32)	1.5 (0.85)	(0.90, 1.90)^***^	2.9 (0.33)	2.3 (0.87)	(−1.67, −0.01)^*^
Sleep disturbances	2.4 (0.70)	1.2 (0.92)	(0.75, 1.65)^***^	2.3 (0.71)	1.6 (0.89)	(−1.23, 0.52)
Daytime dysfunction	1.1 (0.88)	0.5 (0.71)	(−0.01, 1.20)	1.1 (1.05)	0.9 (0.78)	(−1.11, 0.33)

^(1)^Statistical analysis was performed using intent-to-treat (ITT) analysis with SPSS. ^(2)^A paired *t*-test was used to compare differences in the variables between baseline and week 4 in the SZR-ZZC group. ^(3)^A two-sample *t*-test was used to compare differences in the variables between the two groups at week 4; ^*^indicating ^*^ *P* < 0.05; ^**^indicating ^*^ *P* < 0.01; ^***^indicating ^*^ *P* < 0.001. SZR-ZZC: *Suan  Zao  Ren  Tang* + *Zhi  Zi  Chi  Tang*; SD: standard deviation; CI: confidence interval; PSG: polysomnography; TST: total sleep time; LPS: latency to onset of persistent sleep; WASO: wake after sleep onset; SWS: slow wave sleep (stage 3 + stage 4); REM: rapid eye movement sleep; ISI: Severity Index; SAS: Self-Rating Anxiety Scale; PSQI: Pittsburgh Sleep Quality Index.

## References

[1] Ohayon M. M. (2002). Epidemiology of insomnia: what we know and what we still need to learn. *Sleep Medicine Reviews*.

[2] Morin C. M., LeBlanc M., Daley M., Gregoire J. P., Mérette C. (2006). Epidemiology of insomnia: prevalence, self-help treatments, consultations, and determinants of help-seeking behaviors. *Sleep Medicine*.

[3] Neckelmann D., Mykletun A., Dahl A. A. (2007). Chronic insomnia as a risk factor for developing anxiety and depression. *Sleep*.

[4] Wang L. Y., Song Y. H., Li F. (2014). Effects of *Wen Dan tang* on insomnia-related anxiety and levels of the brain-gut peptide ghrelin. *Neural Regeneration Research*.

[5] Sutton E. L. (2014). Psychiatric disorders and sleep issues. *The Medical Clinics of North America*.

[6] Zisapel N. (2012). Drugs for insomnia. *Expert Opinion on Emerging Drugs*.

[7] Warner M., Chen L. H., Makuc D. M. (2009). Increase in fatal poisonings involving opioid analgesics in the United States, 1999–2006. *NCHS Data Brief*.

[8] Lee K.-H., Tsai Y.-T., Lai J.-N., Lin S.-K. (2013). Concurrent use of hypnotic drugs and Chinese herbal medicine therapies among Taiwanese adults with insomnia symptoms: a population-based study. *Evidence-Based Complementary and Alternative Medicine*.

[9] Liu W. J., Hu L. L., Zhang Y. H. (2014). Clinical research on Suanzaoren decoction with Zhizichi decoction treating anxiety-associated insomnia. *Zhejiang Journal of Integrated Traditional Chinese and Western Medicine*.

[10] Bastien C. H., Vallières A., Morin C. M. (2001). Validation of the insomnia severity index as an outcome measure for insomnia research. *Sleep Medicine*.

[11] Bastien C. H., Vallières A., Morin C. M. (2001). Validation of the insomnia severity index as an outcome measure for insomnia research. *Sleep Medicine*.

[12] Chung K.-F., Kan K. K.-K., Yeung W.-F. (2011). Assessing insomnia in adolescents: comparison of insomnia severity index, athens insomnia scale and sleep quality index. *Sleep Medicine*.

[13] Yu D. S. F. (2010). Insomnia Severity Index: psychometric properties with Chinese community-dwelling older people. *Journal of Advanced Nursing*.

[14] Rechtschaffen A., Kales A. (1968). *A Manual of Standardized Terminology: Techniques and Scoring System for Sleep Stages of Human Subjects*.

[15] Liang T., Liu E.-W., Zhong H., Wang B., Shen L.-M., Wu Z.-L. (2008). Reliability and validity of addiction severity index in drug users with methadone maintenance treatment in Guizhou Province, China. *Biomedical and Environmental Sciences*.

[16] Li A. (2009). Analyses on the rate and epidemic characteristics of anxiety and depression among cancer patients in yangpu district in shanghai. *Asian Pacific Journal of Cancer Prevention*.

[17] Buysse D. J., Reynolds C. F., Monk T. H., Berman S. R., Kupfer D. J. (1989). The Pittsburgh Sleep Quality Index: a new instrument for psychiatric practice and research. *Psychiatry Research*.

[18] Backhaus J., Junghanns K., Broocks A., Riemann D., Hohagen F. (2002). Test-retest reliability and validity of the Pittsburgh Sleep Quality Index in primary insomnia. *Journal of Psychosomatic Research*.

[19] Sethi P. K., Khandelwal D. C. (2005). Zolpidem at supratherapeutic doses can cause drug abuse, dependence and withdrawal seizure. *The Journal of Association of Physicians of India*.

[20] Victorri-Vigneau C., Dailly E., Veyrac G., Jolliet P. (2007). Evidence of zolpidem abuse and dependence: results of the French Centre for Evaluation and Information on Pharmacodependence (CEIP) network survey. *British Journal of Clinical Pharmacology*.

[21] Yang Y. H., Lai J. N., Lee C. H., Wang J. D., Chen P. C. (2011). Increased risk of hospitalization related to motor vehicle accidents among people taking zolpidem: a case-crossover study. *Journal of Epidemiology*.

[22] Sakamoto T., Uchimura N., Mukai M., Mizuma H., Shirakawa S.-I., Nakazawa Y. (1998). Efficacy of L-846 in patients with insomnia: evaluation by polysomnography. *Psychiatry and Clinical Neurosciences*.

[23] Zhang S., Zhao L., Wang H. (2013). Efficacy of modified LiuJunZi decoction on functional dyspepsia of spleen-deficiency and qi-stagnation syndrome: a randomized controlled trial. *BMC Complementary and Alternative Medicine*.

[24] Li Q., Yang G.-Y., Liu J.-P. (2013). Syndrome differentiation in Chinese herbal medicine for irritable bowel syndrome: a literature review of randomized trials. *Evidence-Based Complementary and Alternative Medicine*.

[25] Yeh C.-H., Arnold C. K., Chen Y.-H., Lai J.-N. (2011). *Suan Zao Ren Tang* as an original treatment for sleep difficulty in climacteric women: a prospective clinical observation. *Evidence-Based Complementary and Alternative Medicine*.

[26] Chen F. P., Jong M. S., Chen Y. C., et al (2011). Prescriptions of Chinese herbal medicines for insomnia in Taiwan during 2002. *Evidence-Based Complementary and Alternative Medicine*.

[27] Yang B., Zhang A., Sun H. (2012). Metabolomic study of insomnia and intervention effects of Suanzaoren decoction using ultra-performance liquid-chromatography/electrospray-ionization synapt high-definition mass spectrometry. *Journal of Pharmaceutical and Biomedical Analysis*.

[28] Yeung W.-F., Chung K.-F., Man-Ki Poon M. (2012). Chinese herbal medicine for insomnia: a systematic review of randomized controlled trials. *Sleep Medicine Reviews*.

[29] Chen C. Y. C., Chen Y. F., Wu C. H., Tsai H. Y. (2008). What is the effective component in suanzaoren decoction for curing insomnia? Discovery by virtual screening and molecular dynamic simulation. *Journal of Biomolecular Structure and Dynamics*.

[30] Qu K., Dai J., Zhao L. (2013). A sensitive liquid chromatographic-mass spectrometric method for simultaneous quantification of six iridoid glycosides from Zhi-zi-chi Decoction in rat plasma and its application to a pharmacokinetic study. *Journal of Pharmaceutical and Biomedical Analysis*.

[31] Wu T.-X., Shang H.-C., Bian Z.-X. (2009). Randomized controlled pragmatic trial: concept, design, practice. *Chinese Journal of Evidence-Based Medicine*.

